# Headspace Solid Phase Micro Extraction Gas Chromatographic Determination of Fenthion in Human Serum

**DOI:** 10.3390/ijms9050906

**Published:** 2008-05-23

**Authors:** Konstantinos M. Kasiotis, Helen Souki, Angelos N. Tsakirakis, Haris Carageorgiou, Spiridon A. Theotokatos, Serkos A. Haroutounian, Kyriaki Machera

**Affiliations:** 1 Benaki Phytopathological Institute, Laboratory of Pesticides Toxicology, 8 St. Delta Street, Kifissia 14561, Athens, Greece; E-mail: kasiotk@otenet.gr; k.machera@bpi.gr; 2 Pharmacology Department, Medical School, University of Athens, 115 27, Athens, Greece; E-mail: xkarag@med.uoa.gr; 3 Agroindustrial Robola Growers Cooperative of Kefalllinia, Omala, 28100 Kefallinia, Greece; E-mail: robola@altecnet.gr; 4 Chemistry Laboratory, Agricultural University of Athens, Iera Odos 75, 118 55, Athens, Greece; E-mail: sehar@aua.gr

**Keywords:** Insecticide residue, olive, fenthion, headspace, SPME, GC/MS, metabolites

## Abstract

A simple and effective analytical procedure was developed for the determination of fenthion residues in human serum samples. The sample treatment was performed using the headspace solid-phase micro extraction with polyacrylate fiber, which has the advantage to require low amount of serum (1 mL) without tedious pre-treatment. The quantification of fenthion was carried out by gas chromatography-mass spectrometry and the recoveries ranged from 79 to 104% at two spiking levels for 6 replicates. Detection and quantification limits were calculated as 1.51 and 4.54 ng/mL of serum respectively. Two fenthion metabolites fenoxon and fenthion–sulfoxide were also identified.

## 1. Introduction

**Scheme 1.**
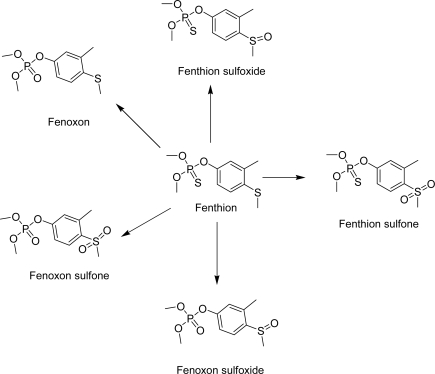
Fenthion metabolites derived from enzymatic desulfuration (oxon analogues) and photolytic process (thiooxidation products).

Organophosphorous insecticides such as dimethoate and fenthion constitute the most commonly applied means to protect olive fruits from *Dacus oleae*, which is the major pest of olive fruits in the Mediterranean region. In Greece, fenthion was used as the molecule of choice to control the action of *Dacus oleae* and was applied for almost 25 years in olive groves, before its banning on June of 2007. Thus, there is a considerable interest in fenthion and its metabolites residue (Scheme 1) assays in human samples [1].

Many multiresidue procedures which utilize a variety of cleanup techniques and diverse detection methods have been developed for the determination of organophosphorous insecticide residues in olive oils [2]. Most of these methods include laborious sample preparation procedures, such as the liquid-liquid (LLE) or solid phase (SPE) extractions.

The proposed method herein is a simple and time effective procedure for the direct determination of fenthion that combines the headspace solid-phase microextraction (HS-SPME) technique with the gas chromatography system (GC-MS). This approach has recently gained a considerable interest and its application in the assessment of organic molecules in human biological samples. Simplicity constitutes its main advantage, since the tedious sample extraction and/or derivatization steps of other methods have been circumvented, while the potential matrix effect was minimized [3–5]. On the other hand, because fenthion is not a thermally labile organic compound – is stable up to 210°C [6] – the HS-SPME methodology was applied under parallel heating, enhancing thus its extraction-absorption potentials. Finally, two metabolic products of fenthion using a GC-MS system on Single Ion Monitoring (SIM) mode have been identified.

## 2. Results and Discussion

### Development of HS-SPME procedure

The procedure presented has been based on a previously reported solid phase microextraction method for the analysis of organophosphorous (OP) insecticides in fruits [7] and in the present study it was adapted to biological samples. Thus, the applied method is a combination of the above-mentioned SPE microextraction method and a previously described procedure for the determination of another OP insecticide, dimethoate, in human biological samples by gas chromatographic analysis [8]. Based on the above a straightforward method with the use of polyacrylate (PA) fiber has been developed, which does not require the addition of any organic solvent or prolonged analyte desorption times. The best extraction was determined at 20 min from several trials at different time points (10, 15, 20, 25, 35, 40, 60 and 90 min). The developed methodology is simple and cost effective involving two main steps, i.e the sample heating step and the subsequent direct SPME fiber headspace adsorption exposure.

### Assessment of applicators exposure

The results of fenthion concentration assessments in human serum samples – expressed as μg of fenthion per mL of human serum – are summarized in Tables 1 and 2. Furthermore, the total fenthion amount per applicant was estimated assuming an average of blood volume of 5L for male addults. No fenthion contamination was detected in non-exposed human volunteers’ samples.

### Linearity

**Figure 1.**
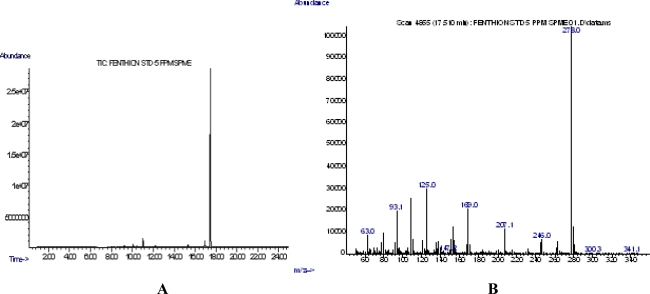
**A)** GC Chromatogram of a 5 ppm fenthion solution. **B)** MS fragmentation pattern of fenthion (5 ppm)

Fenthion was determined (retention time, 17.510 min, Figure 1A) using the HS-SPME-GC-MS methodology, based on the characteristic for fenthion ions at m/z 278 and 169 (Figure 1B). In each case, both SIM and full scan methods were applied producing good response linearity for concentrations ranging from 0.05 to 15 ng/mL, with a correlation coefficient of r^2^>0.997.

### Limit of Detection (LOD)–Limit of Quantification (LOQ)

The limits of detection (LOD) and quantification (LOQ) were determined via statistical calculations using a calibration plot (*y = 31253x+1220*) of fenthion which was established at concentration levels close to the expected LOD *i.e*. at 0.00005, 0.0005, 0.001 μg/mL. The LOD was defined as 3.3(*Sy/x*)/*a* and the LOQ as 10(*Sy/x*)/*a*, where *Sy/x* represents the residual standard deviation and *a* is the slope of the calibration plot. Thus, LOD and LOQ were calculated at 1.51 and 4.54 ng/mL of serum respectively.

### Recovery study

The recovery study was performed at two different concentration levels on 6 replicates which were spiked with fenthion. Satisfactory results were obtained in both cases, with recoveries ranging from 79 to 104%. RSDs for low concentrations were between 1.4 – 7.5 %, which is indicative of the methods precision. RSDs for high concentrations were determined between 2.4 – 8.1 %.

**Figure 2.**
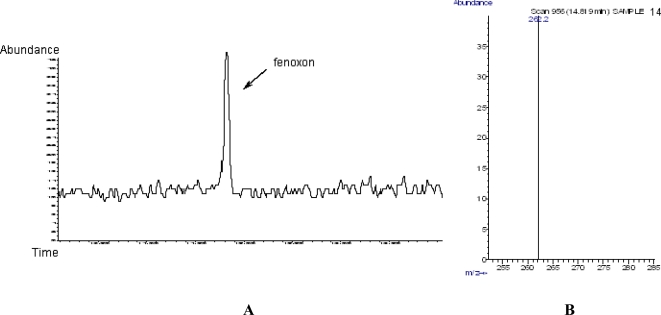
**A)** GC chromatogram of fenoxon (retention time 14.819 min). **B)** MS Characteristic ion of fenoxon (262.2 amu).

Furthermore, two fenthion metabolites were also identified using the GC-MS-SIM method. More specifically, fenoxon was detected (retention time 14.819 min, Figure 2A) and identified via its characteristic ion at 262,2 amu (Figure 2B).

**Figure 3.**
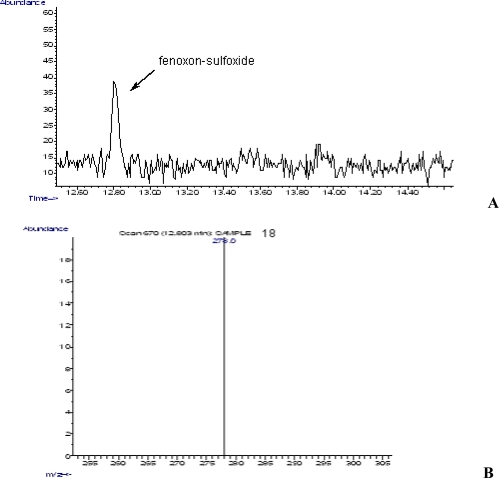
**A)** GC chromatogram of fenoxon-sulfoxide (retention time, 12.803 min). **B)** MS Characteristic ion of fenoxon-sulfoxide (278.0 amu).

The second metabolite detected was fenthion-sulfoxide (retention time 12.803 min, Figure 3A) and displayed a characteristic ion at 278 amu (Figure 3B).

## 3. Experimental Section

### Reference Item

The reference item of fenthion (purity 97.7%) was purchased from Riedel de Häen.

### Operators

A total of 25 operators with adequate experience in spraying pesticides were selected. They were given the study details, procedures, safety precautions and their obligations throughout the monitoring phase. All the operators signed a consent form to express their willingness to participate in the study. Neither of the pesticide applicators was involved in spraying activities, for at least two months, before fenthion applications and the respective monitoring [9].

The applications were carried out in olive groves in Agrinio, Etoloakarnania, Greece and the application practice was the knapsack sprayer.

### Blood samples handling

Before the start of the study blood was taken from volunteers for normal blood tests for the assessment of their physical condition. At the end of the application period (5 days), 10 mL of blood samples were taken in Wasserman tubes. The serum was separated by centrifiguration of the samples for 5 min at 3.500 rpm in a Hettich Rotofix 32 centrifuge and transferred to a deep freezer, located in storage near to the application area. Following the termination of the sampling period the samples were transported in dry ice to the Laboratory of Pesticides Toxicology at Benaki Phyropathological Institute where they were stored in deep freezers until analysis. An equal number of blood samples was collected from non-exposed volunteers, transported and stored as previously mentioned.

### Apparatus and Chromatography

#### GC-MS

Analysis was carried out on an Agilent 6890N chromatograph equipped with a split splitless injector and a 5975B inert XL EI/CI MSD (Agilent Technologies) connected to MSDChemStation G1701 DA MSD software, version D.03.00.611. The capillary column was a DB-5MS (30m ×0.25mm ×1.0 μm) with 5% diphenyl–95% dimethylsiloxane. The injector and detector were operated at 200°C and 280°C, respectively. The sample (1μL) was injected into the pulsed splitless mode and the oven temperature was programmed as follows: 60 °C for 1 min, raised to 220°C (20°C /min), raised to 227°C (2°C /min) for 3 min, raised to 265°C (20°C /min) for 1 min, raised to 270°C (1°C /min) and to 280 °C (20°C /min) for 1 min. Helium was the carrier gas (1.8 mL/min) and nitrogen (30 mL/min) the make-up gas.

### Development of HS-SPME procedure

SPME holders and fibers were obtained from Supelco and polyacrylate (PA, 85 μm) fiber was used. In particular, 1 mL of human serum was shaken in a vortex mixer for 1 min, transferred into a headspace vial (9 mL volume from Alltech, Alltech Ass. Deerfield, IL) and placed in an aluminium block heater. After 20 min of preheating period at 70°C with simultaneous stirring, the SPME needle pierced the vial septum and the fiber was exposed in the headspace of the serum solution. Sampling was performed for additional 20 min at 140°C and finally the needle was removed from the vial and injected into the heated injection port of the gas chromatograph. Desorption was allowed for 5 min.

The same procedure was followed for the control samples for the preparation of the calibration curve. For the establishment of the calibration curve, 1 mL of fenthion from 0.05 to 15 ng/mL, was placed in a headspace vial (9 mL volume from Alltech, Alltech Ass. Deerfield, IL) and evaporated to dryness by a gentle stream of nitrogen. Afterwards, 1 mL of control-blank samples of serum was transferred via a syringe to the headspace. Following the mentioned procedure the needle was exposed in the headspace vial and injected into the heated injection port of the gas chromatograph for desorption for 5 min.

## 4. Conclusions

Headspace SPME technique proved to be efficient for the determination of fenthion and the identification of two of its metabolites in human serum samples. It is easy to handle, solvent free and has the advantage of cost effectiveness. The matrix (serum) does not inhibit the adsorption efficacy, which is evident by the satisfactory recoveries observed. On the whole the described procedure is efficient and as a useful tool can be potentially applied for the determination of other insecticides of the organophosphorous group.

## Figures and Tables

**Table 1.** Exposure of Applicators (**1–14**) to fenthion.

Operator	1	2	3	4	5	6	7	8	9	10	11	12	13	14
**Serum**	1 mL													
**Body Weight (kg)**	70	81	82	68	65	75	86	78	90	71	68	80	65	70
**Conc. μg/mL**	0.42	5.23	7.02	1.61	3.88	2.46	2.26	2.85	7.20	5.50	0.87	1.14	0.90	1.27
	E-04	E-04	E-04	E-04	E-04	E-04	E-04	E-04	E-04	E-04	E-04	E-04	E-04	E-04
**Total Amount in Serum (μg).^A^**	0.21	2.62	3.51	0.81	1.94	1.23	1.13	1.43	3.60	2.75	0.44	0.57	0.45	0.64

A Table note: Extrapolation to the volume of 5L (5000mL blood).

**Table 2.** Exposure of Applicators (**15–25**) to fenthion.

Operator	15	16	17	18	19	20	21	22	23	24	25
**Serum**	1 mL										
**Body Weight (kg)**	67	87	64	70	81	73	76	85	79	69	82
**Conc. μg/mL**	7.13	2.12	2.31	10.05	0.73	1.67	2.67	5.67	0.19	4.71	0.87
	E-04	E-04	E-04	E-04	E-04	E-04	E-04	E-04	E-04	E-04	E-04
**Amount in Serum (μg).A**	3.57	1.06	1.16	5.03	0.37	0.84	1.34	2.84	0.10	2.36	0.44

A Table note: Extrapolation to the volume of 5L (5000mL blood).
